# Non-invasive diagnosis of deep vein thrombosis from ultrasound imaging with machine learning

**DOI:** 10.1038/s41746-021-00503-7

**Published:** 2021-09-15

**Authors:** Bernhard Kainz, Mattias P. Heinrich, Antonios Makropoulos, Jonas Oppenheimer, Ramin Mandegaran, Shrinivasan Sankar, Christopher Deane, Sven Mischkewitz, Fouad Al-Noor, Andrew C. Rawdin, Andreas Ruttloff, Matthew D. Stevenson, Peter Klein-Weigel, Nicola Curry

**Affiliations:** 1 ThinkSono Ltd, London, UK; 2 Imperial College London, London, UK; 3 FAU Erlangen-Nürnberg, Erlangen, Germany; 4 King’s College London, London, UK; 5 University of Lübeck, Institute of Medical Informatics, Lübeck, Germany; 6 ThinkSono GmbH, Potsdam, Germany; 7 Central Alberta Medical Imaging Services, Red Deer, AB Canada; 8 Oxford Haemophilia and Thrombosis Centre, Headington, UK; 9 The University of Sheffield, School of Health and Related Research, Sheffield, UK; 10 Clinic of Angiology - Interdisciplinary Center of Vascular Medicine, Potsdam, Germany

**Keywords:** Diagnosis, Haematological diseases

## Abstract

Deep vein thrombosis (DVT) is a blood clot most commonly found in the leg, which can lead to fatal pulmonary embolism (PE). Compression ultrasound of the legs is the diagnostic gold standard, leading to a definitive diagnosis. However, many patients with possible symptoms are not found to have a DVT, resulting in long referral waiting times for patients and a large clinical burden for specialists. Thus, diagnosis at the point of care by non-specialists is desired. We collect images in a pre-clinical study and investigate a deep learning approach for the automatic interpretation of compression ultrasound images. Our method provides guidance for free-hand ultrasound and aids non-specialists in detecting DVT. We train a deep learning algorithm on ultrasound videos from 255 volunteers and evaluate on a sample size of 53 prospectively enrolled patients from an NHS DVT diagnostic clinic and 30 prospectively enrolled patients from a German DVT clinic. Algorithmic DVT diagnosis performance results in a sensitivity within a 95% CI range of (0.82, 0.94), specificity of (0.70, 0.82), a positive predictive value of (0.65, 0.89), and a negative predictive value of (0.99, 1.00) when compared to the clinical gold standard. To assess the potential benefits of this technology in healthcare we evaluate the entire clinical DVT decision algorithm and provide cost analysis when integrating our approach into diagnostic pathways for DVT. Our approach is estimated to generate a positive net monetary benefit at costs up to £72 to £175 per software-supported examination, assuming a willingness to pay of £20,000/QALY.

## Introduction

Venous thromboembolism (VTE) is associated with a major global burden of disease. Worldwide, the incidence of VTE is 1–3 per 1000 individuals, rising to 2–7 per 1000 in individuals aged over 70 years, and 3–12 per 1000 in those over 80 years^1^. VTE, deep vein thrombosis (DVT) and pulmonary embolus (PE) are the leading cause of hospital-related disability-adjusted life years lost^2^.

Using these estimates, and using the most conservative incidence figure, globally at least 7.7 million people will require investigation for VTE every year. An ageing population across many countries will lead to a greater health burden, particularly in middle- and low-income countries where early death from infection is decreasing. Mortality from VTE is common, a European study estimated 534,000 deaths per year^3^ and a similar study in the US reported 300,000 deaths per year^4^. DVT has a high level of morbidity. 30–50% of the surviving patients develop long-term symptoms in their affected leg (post-thrombotic syndrome)^5^.

In high-income countries, the routine practice to diagnose patients after a positive D-dimer blood test and an indicative evaluation using the Wells score^6^ is to confirm or rule out a suspected DVT with a two- or three-point ultrasound scan. Ultrasound scans are most commonly performed in a radiology or cardiovascular department of a hospital by a highly trained radiographer/radiologist.

Currently, no reliable test is available that can be used in a general healthcare setting (GP practice, community hospital, on a hospital ward) or be used remotely at the point of care (nursing home, patient’s home). Between 85 and 90% of patients presenting to their GP in high-income countries with a suspected DVT will be investigated only to find no evidence of a thrombus^5^. Many patients will receive unnecessary anticoagulants with numerous potential side-effects through an often-painful subcutaneous injection whilst waiting more than the recommended four hours for their scan. Safely negating this wait would improve patient satisfaction, reduce the burden of high-risk treatment (anticoagulants confer haemorrhagic complication risks) and discount healthcare costs. Rapid diagnosis is known to improve compliance to regulatory guidelines that state DVT should be diagnosed within 24 h^6–8^. Clinical evidence that DVT examinations using ultrasound can be performed by nurses has been shown^9–11^. However, confidence in acquiring ultrasound images is generally low because of the required image interpretation skills and liability concerns, which inhibits wide-scale adoption of such approaches. In this study we evaluate if Machine Learning (ML) technology can provide anatomical image acquisition guidance and point of care diagnostic support. Such ML technology is currently often summarised as Artificial Intelligence (AI) support systems.

ML technology has previously been explored in the context of VTE, with several studies having shown the potential for ML clinical decision support systems (CDSS) to add incremental value in improving VTE risk stratification of patients. Most of these proposed CDSS are predominantly based upon the Wells criteria^12^, whilst others are more complex, taking into consideration a broader range of clinical risk factors for VTE as identified in the Caprini model (35 discrete clinical risk factors)^13,14^. However, to the best of our knowledge, no prior study has shown the potential benefit of ML to aid in the image-based diagnosis of DVT using ultrasound. Our hypothesis is that ML technology can complement the clinical pathway and provide non-specialists with the necessary confidence and skills to perform ultrasound DVT screening autonomously. Early modelling has been undertaken to assess the potential cost-effectiveness of such an approach.

## Results

### Study participation

#### External Validation Set 1 (EVS1)

124 patients who presented to the Oxford Haemophilia and Thrombosis Centre, Oxford, UK, with symptoms suggestive of DVT were approached for inclusion into this study. Compression ultrasound has been performed according to the standard practice, without software guidance. Patients have first been scanned as part of the standard pathway with various scanners, followed by another scan using a provided Philips Lumify probe with screen recording software.

The recorded screen capture videos have been curated to a data set that is similar in nature to one as it would have been acquired with AutoDVT software guidance.

**Fig. 1:**
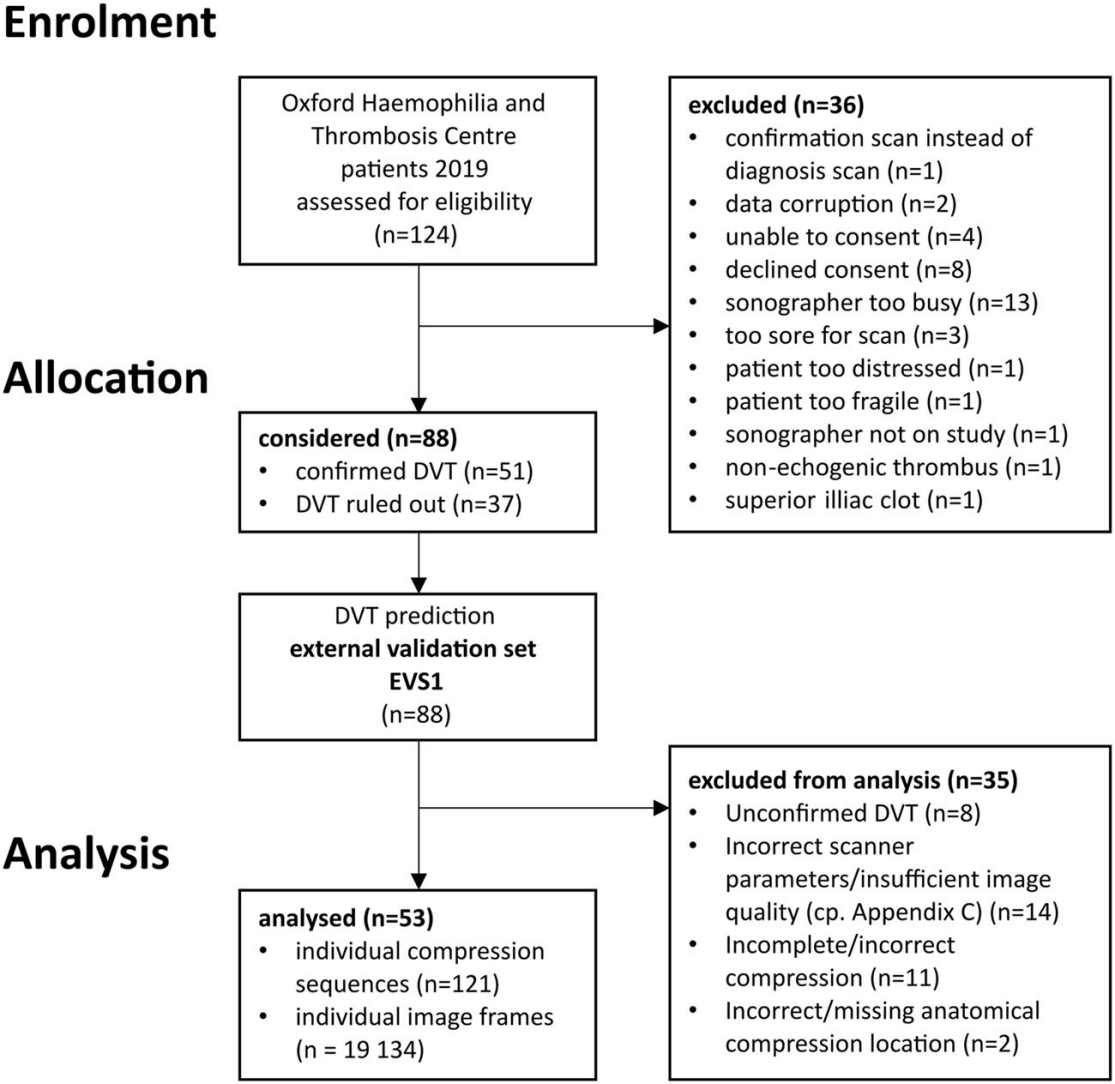
Consort diagram for study enrolment. Allocation, and analysis in External Validation Set 1 (EVS1).

Thirty-six patients have been excluded during the enrolment phase for various reasons as summarised in the Consort Diagram in Fig. 1. Two patients with confirmed DVT have been excluded due to imaging conditions that are not covered by the standard compression ultrasound DVT protocol (non-echogenic thrombus and superior thrombosis in the iliac vein). Control participants had no DVT based on comprehensive clinical and laboratory testing performed under the supervision of and interpreted by a haematologist. This results in a data set comprising of 88 eligible patients. An overview of patient characteristics in this clinic’s database is given in Table 1.

**Table 1:** General population overview for model training and external validation set.

Age [years]	64.2 ± 17.7
Wells score	1.67 ± 1.12
D-Dimer [micrograms/litre fibrinogen equivalent units]	1870 ± 3070 (one-sided)
Male [%]	46.4
Female [%]	53.5
Not stated [%]	0.1
proximal DVT diagnosis [%]	7.1
Demographics	
Asian - Any Other Asian Background	0.49%
Asian or Asian British - Indian	0.52%
Asian or Asian British - Pakistani	1.40%
Black - Any Other Black Background	0.29%
Black or Black British - African	0.59%
Black or Black British - Caribbean	0.29%
Mixed - Any Other Mixed Background	0.20%
Mixed - White and Asian	0.10%
Mixed - White and Black African	0.10%
Mixed - White and Black Caribbean	0.13%
Other - Any Other Ethnic Group	0.39%
Other - Chinese	0.10%
Other - Not Known	0.39%
Other - Not Stated	15.31%
White - Any Other White Background	3.58%
White - British	74.85%
White - Irish	0.65%
Not recorded	0.62%

It was specified that all examinations that were not performed according to the standard implemented in our study design should be omitted, thus, secondary exclusion criteria must be applied. Hence, 35 patients (17 DVT positive, 18 DVT negative) have been further excluded during the analysis phase, due to, radiologist/haematologist confirmed, incorrect/incomplete compression (11), compression on incorrect/missing anatomical location (2), incorrect scanner parameters evaluated by 10-point expert image quality scoring (14). The remaining sequences from positive DVT patients may not include a video clip that confirms the positive DVT. Thus, further eight sequences belonging to a positive DVT case have been excluded. Of the remaining 53 patients 34 patients are DVT positive and 19 DVT negative, confirmed by to the current clinical pathway. This results in 121 individual compression sequences conforming the standard implemented in AutoDVT on defined anatomical vessel locations in these patients. EVS1 was drawn from the general population of 2041 patients with suspected DVT from the Haemophilia and Thrombosis Centre at University of Oxford. The characteristics of the entire population during the year 2019 is summarised in Table 1. The ethical approval in place did not allow for the collection of these characteristics for the individual patients that have been enrolled in this study.

#### External Validation Set (EVS2)

Thirty patients with suspected DVT have been recruited from the Clinic of Angiology, Ernst von Bergmann Klinikum, Potsdam, Germany. Four of them were clinically confirmed DVT positive. In contrast to EVS1, EVS2 used AutoDVT during acquisition. Landmarks and compressions have been proposed automatically by the software, however, users can always override suggestions by the ML model irrespective of the validity of the compression. A vascular technician with experience in carotid ultrasound and peripheral arterial cw-Doppler ultrasound, but without any experience in venous compression ultrasound performed the scans and compressions. No patients have been excluded in this pilot study since data suitability for automatic image processing was ensured by the prospective use of AutoDVT during image acquisition and guidance. Validation compression ultrasound was performed by an experienced angiologist performing at least 1000 duplex and compression ultrasound examinations per year.

### Algorithm performance on the internal validation set

**Fig. 2:**
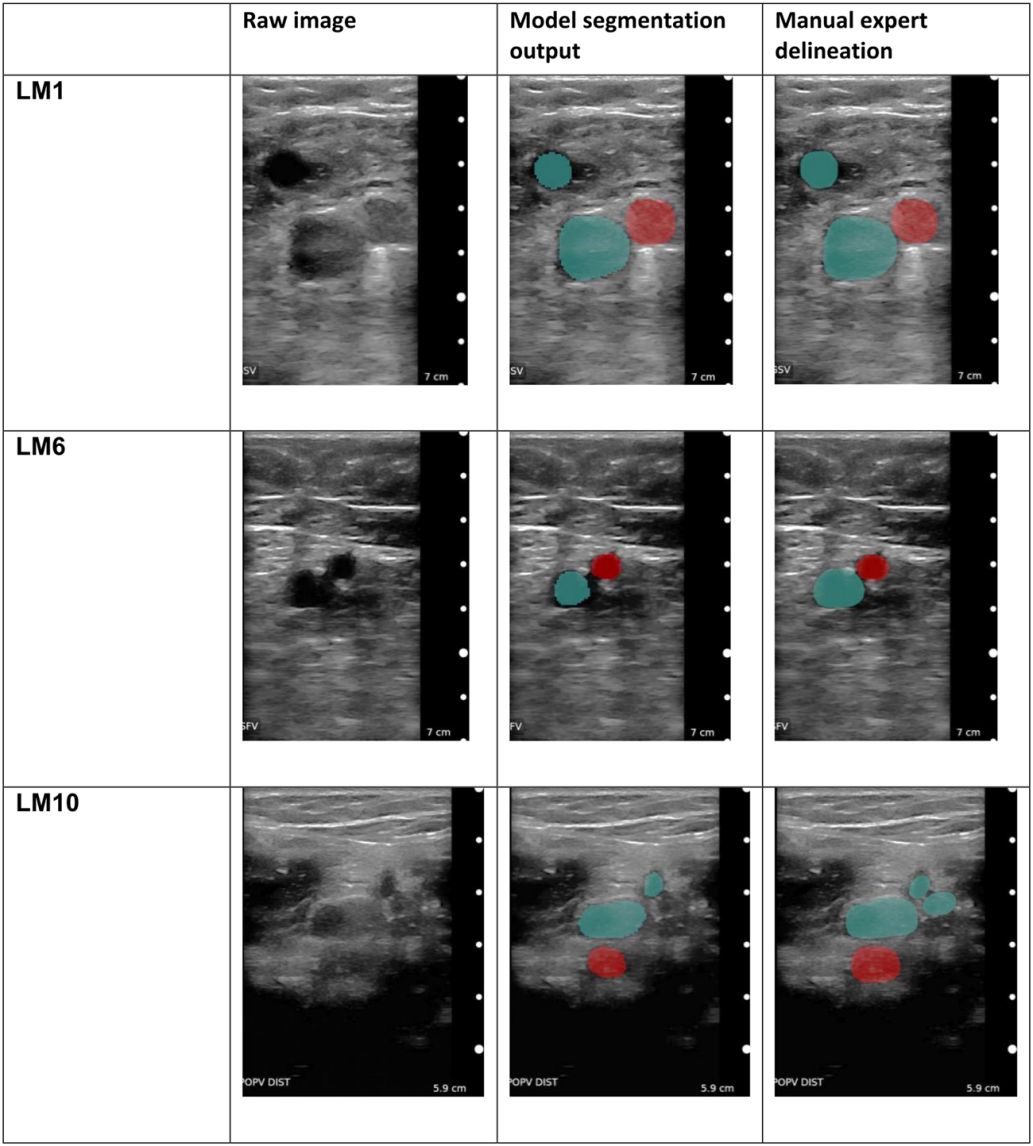
Qualitative example images for our model’s segmentation performance. The segmentation is robust throughout compressions. The vein area is evaluated for complete compressibility to exclude DVT. Device: Clarius L7 (2017).

Figure 2 shows qualitative examples for the segmentation output of our method. Table 2 shows quantitative results for the anatomical landmark detection task; Table 3 for the vessel compression task and Table 4 regarding segmentation performance. Common image evaluation metrics, Sørensen-Dice Coefficient (Eq. (6)) for segmentation results and F1-score (Eq. (5)) for anatomical landmark discrimination and categorical vessel compression analysis, are used for quantitative evaluation.

**Table 2:** Quantitative results for the landmark detection task of the used models.

	Background, no landmark	LM0-LM1 consolidated	LM2-LM3-LM4-LM5 consolidated	LM6-LM7 consolidated	LM8-LM9-LM10 consolidated
Groin and thigh model	(0.95, 0.96)	(0.74, 0.82)	(0.66, 0.70)	(0.67, 0.73)	..
Knee model	(0.93, 0.94)	..	..	..	(0.72, 0.78)

Evaluation according to Eq. (5) on the internal validation set.

(⋅, ⋅) is the 95% confidence interval range.

Model after 50 training epochs.

**Table 3:** Quantitative results for the vein compression state task of the used models.

	Vein open	Vein closed and fully compressed
Groin and thigh model	(0.86, 0.90)	(0.88, 0.96)
Knee model	(0.78, 0.92)	(0.86, 0.92)

Evaluation according to Eq. (5) on the internal validation set. (⋅, ⋅) is the 95% confidence interval range.

**Table 4:** Quantitative results for the vessel segmentation task of the used models.

	Sørensen–Dice Coefficient			Bounding-box intersection over union
	Background	Artery	Vein	
Groin and thigh model	(0.97, 1.00)	(0.82, 0.88)	(0.63, 0.79)	(0.72, 0.84)
Knee model	(0.97, 1.00)	(0.63, 0.85)	(0.73, 0.83)	(0.72, 0.82)
Metric	Eq. (6)			Eq. (7)

Evaluation according to Eqs. (6) and (7) on the internal validation set.

(⋅, ⋅) is the 95% confidence interval range.

### Algorithm performance on the external validation sets

#### EVS1

**Fig. 3:**
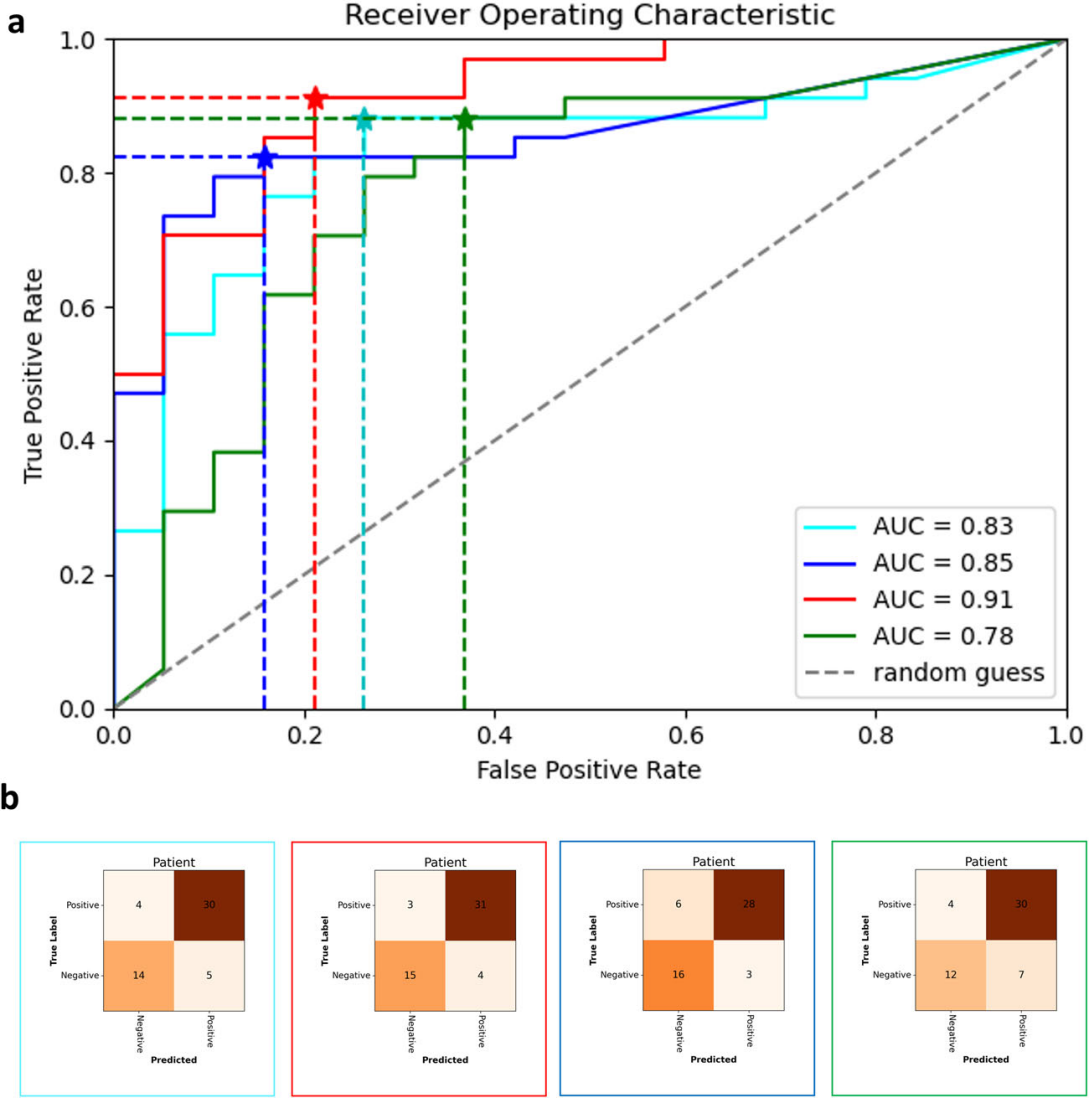
Evaluation results for ESV1 on patient level. Receiver operator characteristics on EVS1 resulting from fourfold cross validation (**a**). Confusion matrices are shown in (**b**) for the optimal threshold (* in (**a**)) in each fold. Frame colours in (**b**) correspond to ROC fold colours in (**a**)). Vessel status is extracted automatically from 53 patients in EVS1 through the combination of fold-specific groin and knee model pairs.

**Fig. 4:**
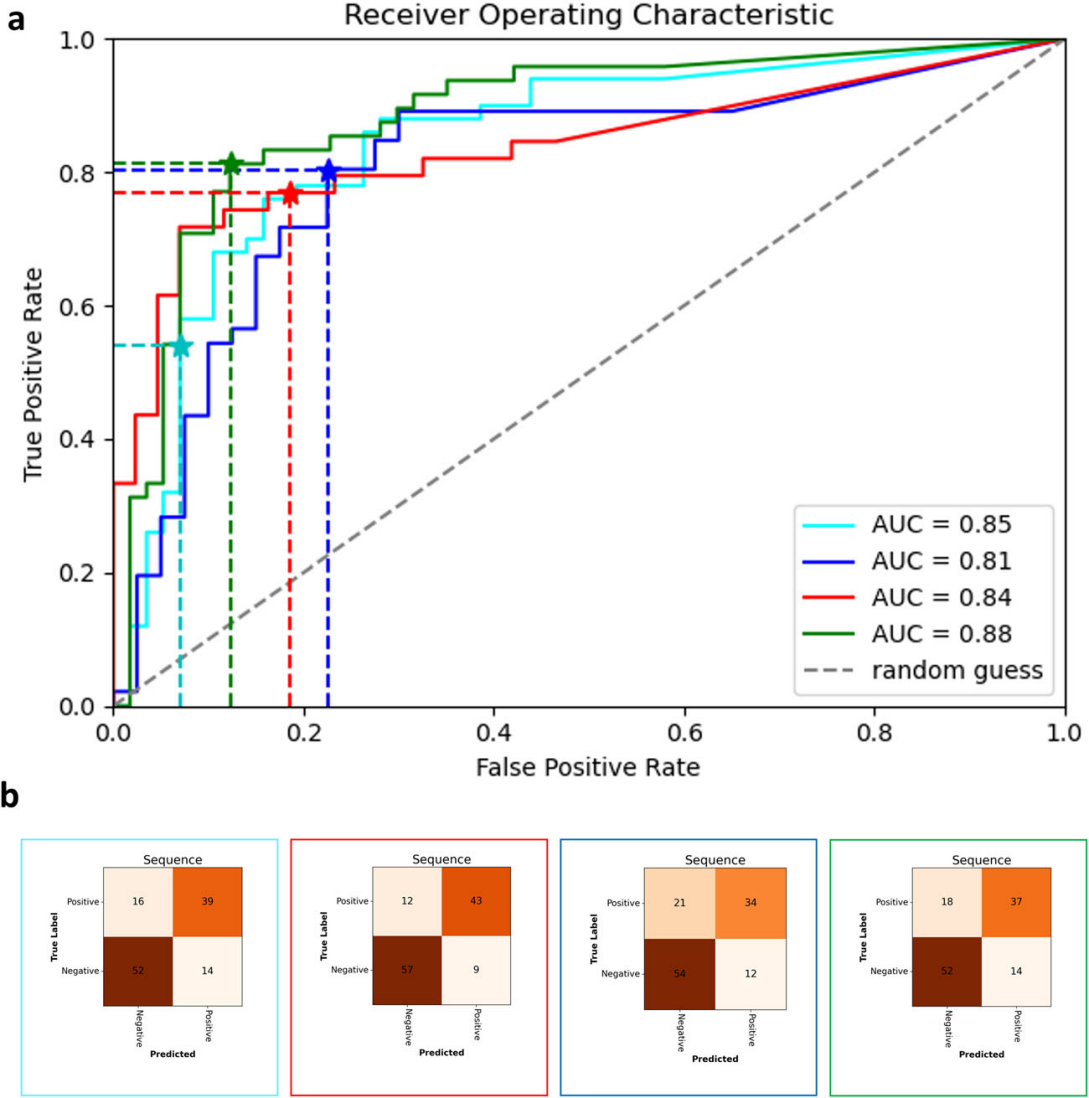
Evaluation results for ESV1 on compression sequence level. Receiver operator characteristics for the correct compression classification per ultrasound sequence/anatomical landmark on EVS1 (**a**). Confusion matrices (**b**) at optimal thresholds (* in (**a**)) per fold. Frame colours correspond to ROC fold colours in (**a**). Vessel status is extracted automatically through the ML models from the 121 available anatomical landmark sequences in EVS1.

**Fig. 5:**
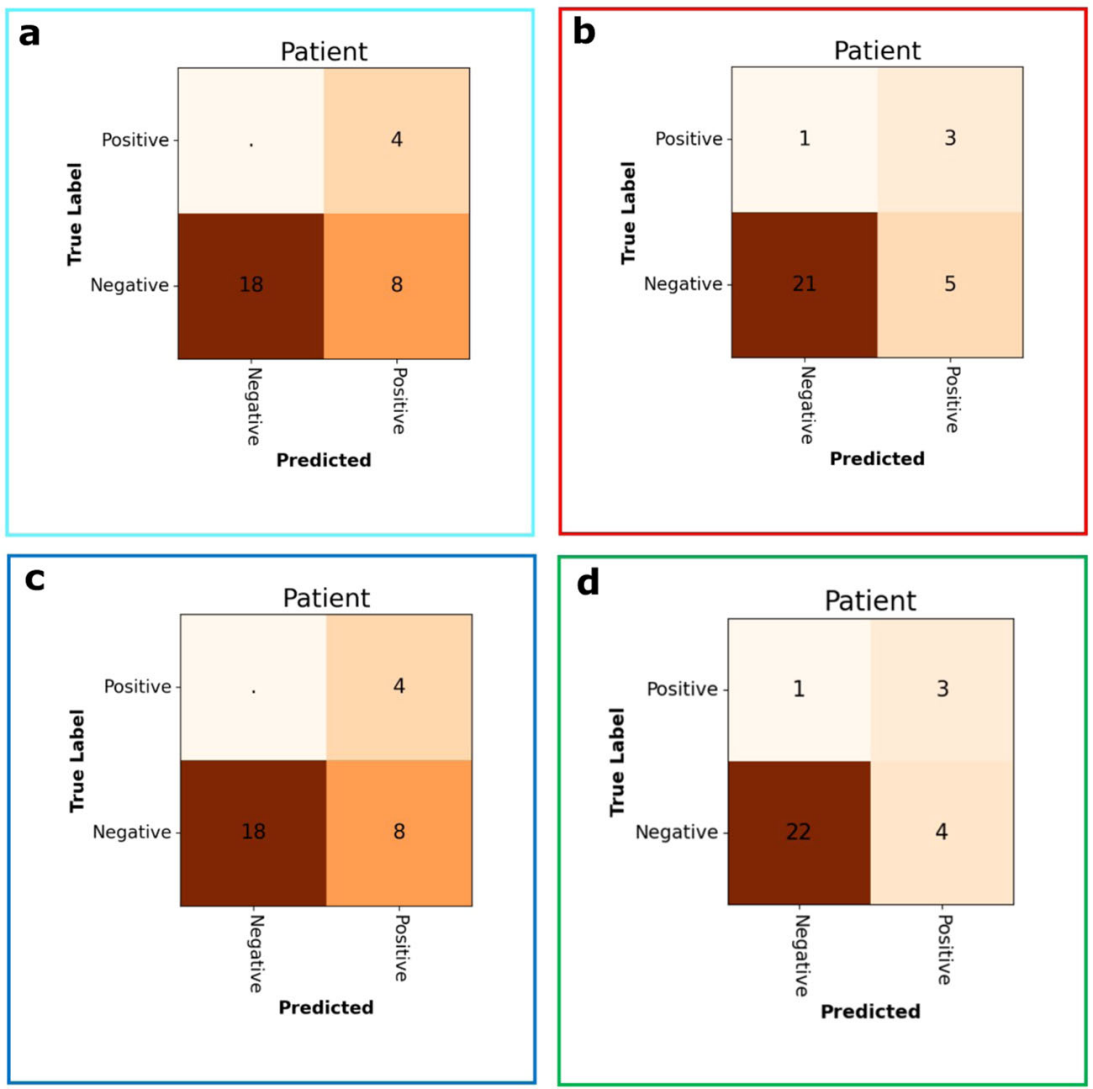
Confusion matrices for EVS2 for each fold. Frame colours correspond to cross-validation folds (**a**–**d**) from Figs. 3 and 4. Vessel status is extracted automatically from all 30 patients in EVS2. Note that this experiment comprises of four DVT positive and 26 DVT negative patients, thus sensitivity/true positive rate is discretised in a ROC curve with only four steps, which makes a plot less meaningful.

Quantitative results on EVS1 are summarised in Table 5. Receiver operator curves are shown together with confusion matrices in Fig. 3 on patient level and Fig. 4 on sequence/anatomical landmark level. Note that these results are based on retrospective analysis of prospectively acquired ultrasound videos without using software guidance. In a perfect prospective setting, AutoDVT guides the operator to acquire images that are well suited for algorithmic evaluation. A setup more akin to the latter paradigm has been tested in the pilot study in EVS2 (Fig. 5).

**Table 5:** Values are expressed between [0,1] intervals and (⋅, ⋅) is the 95% confidence interval range.

Performance metrics for Algorithm predictions	EVS1	EVS2	EVS1 + EVS2
Sensitivity	(0.89, 0.93)	(0.84, 0.92)	(0.82, 0.96)
Specificity	(0.65, 0.83)	(0.69, 0.85)	(0.70, 0.82)
PPV	(0.62, 0.86)	(0.53, 1.00)	(0.65, 0.89)
NPV	(0.98, 0.99)	(0.98, 1.00)	(0.98, 0.99)
Accuracy	(0.78, 0.88)	(0.72, 0.78)	(0.75, 0.83)
AUC	(0.79, 0.91)	(0.71, 0.89)	(0.77, 0.87)

PPV/NPV has been calculated using the population prevalence from Table 1, i.e., 7.1%, (and not the prevalence in the case-control data in EVS1 and EVS2, i.e., up to 62%). This approach was chosen to provide a meaningful numeric value for PPV and NPV for patients^42^.

(⋅, ⋅) is the 95% confidence interval after fourfold cross validation.

EVS1 shows results from *N* = 53 examinations performed with Philips Lumify in Oxford. EVS2, *N* = 30, including four positive DVT cases on Clarius HD (2020). The combined evaluation is shown in column (EVS1 + EVS2).

#### EVS2

Results from 30 DVT-suspected cases (four DVT positive) that have been acquired using the AutoDVT software prospectively in the Clinic of Angiology in Potsdam, Germany, are presented in Table 5 and Fig. 5. We test the same fourfold cross-validation models as used for EVS1 data from Oxford.

### Comparison to a naive black-box classifier

To evaluate the benefits of our ML architecture and explainable image segmentation-based approach over a naïve black box classifier, we have further tested a direct classifier on EVS1 and EVS2. For this experiment, we encoded each image in the available sequences into 128-valued image embeddings using a black box encoder from our previous work^15^ that was trained to predict open or closed vein classification in a healthy volunteer cohort. These embeddings were stacked per sequence and used as an input for a 3-layer 1D-CNN. This CNN subsequently learned binary classification into healthy/pathological sequences from the training set. After 10-fold cross validation this model achieves an area under the receiver operating characteristic curve (AUC) of only (0.65, 0.71) for EVS1 and (0.65, 0.73) for EVS2 on sequence level. On patient level this classifier achieves on EVS1 an AUC of (0.65, 0.77). A Welch’s test comparing predictions between the navie black-box model and our specialised and explainable architecture results in *p* = 0.025 for EVS1, hence, a significant difference can be assumed. EVS2 has only four positive cases, thus overfitting prevents such an analysis for the second eternal validation set.

### Operator skill level robustness

Twenty healthy volunteers have been scanned by three senior medical students representing non-expert users. The students got a brief introduction, performed the entire examination using the Clarius L7 device and reported success and software-specific problems. Each participant performed the examination twice in succession on the same five landmarks (guided by AutoDVT). The average time needed for an examination was 6:16 min (median: 3:47 min) during the first run and 5:24 min (median: 4:17 min) during the second run. 154 out of 200 (75.7%) recorded guided landmark approaches and compression sequences were reported successful. Unsuccessful compression attempts were repeated until deemed successful by AutoDVT or a maximum of three attempts.

### Cost effectiveness

**Fig. 6:**
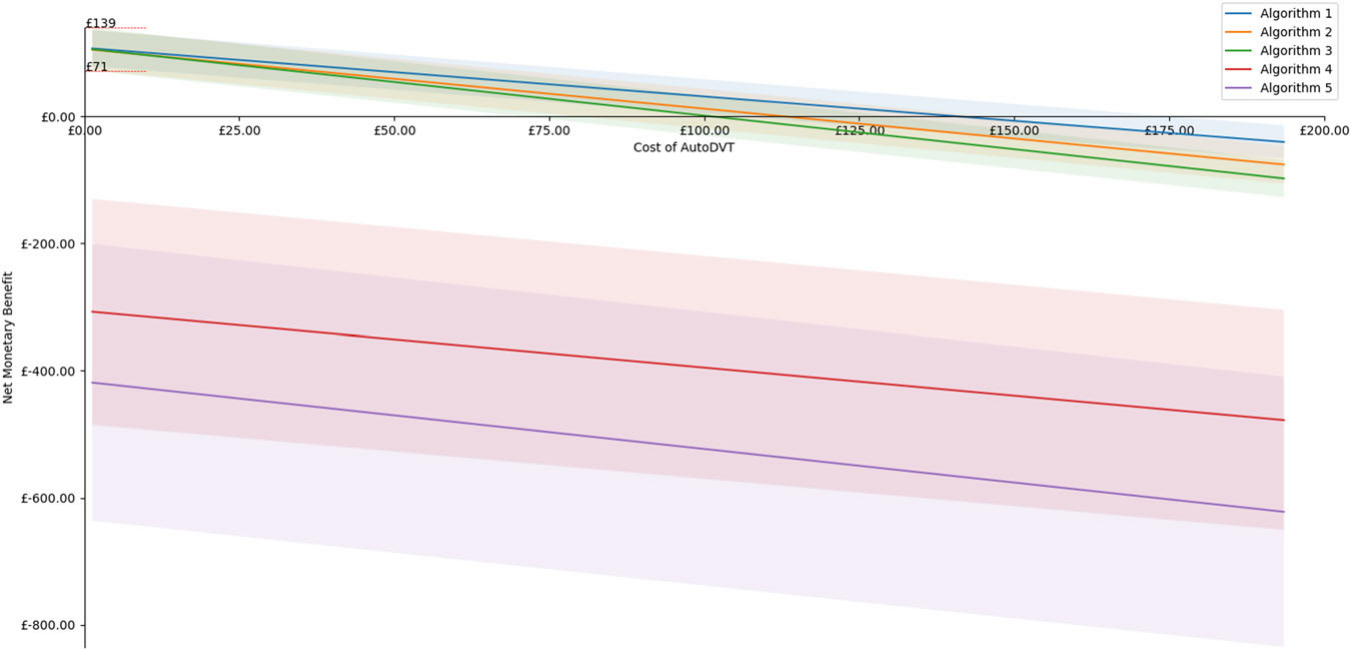
Costs of the guidance tool vs. net monetary benefit (NMB) per examination when implementing ML-guided DVT diagnostics into clinical diagnostic pathways. The NMB has been simulated with a deterministic model for each of the diagnostic algorithm variants in Fig. 11 at the mean (solid line) and the 95 CI interval (shaded area) from Table 5 to show possible optimistic and pessimistic scenarios. The red lines on the y-axis mark the maximal attainable NMB range when examination costs are zero.

D-Dimer plus ultrasound confirmatory scan for DVT diagnostic (Fig. 11a) is currently costed at £92–£97^16^ (Table 14) in the UK NHS. Using the sensitivity and the specificity ranges from Table 5 (EVS1 + EVS2), a maximum positive net monetary benefit (NMB) between £71–£139 per ML-guided examination can be achieved when AutoDVT is integrated into clinical pathways according to Fig. 11. We assume a willingness to pay of £20,000 per QALY^6,17^. Figure 6 shows how the NMB changes with different prices for an ML-guided examination, considering the different diagnostic algorithm variants in Fig. 11. Accuracy versus costs is compared in Table 6.

**Table 6:** Summary of results from the variants in Fig. 11 comparing the calculated total costs per patient pathway and QALYs to the algorithm accuracy.

	Total costs per diagnostic pathway	Total QALYs	True positive likelihood	False positive likelihood	True negative likelihood	False negative likelihood
No software support	£300	11.57	0.10	0.06	0.84	0.00
Algorithm 1	£230–£244	11.57	0.09	0.01–0.02	0.88–0.89	0.01
Algorithm 2	£237–£254	11.57	0.09	0.01–0.02	0.88–0.89	0.01
Algorithm 3	£240–£259	11.57	0.09	0.01–0.02	0.88–0.89	0.01
Algorithm 4	£393–£510	11.56	0.10	0.19–0.30	0.60–0.71	0.00
Algorithm 5	£444–£590	11.56	0.10	0.23–0.37	0.53–0.67	0.00

Assumed population statistics according to DVT prevalence in Table 1.

## Discussion

This study provides a proof of concept that ML-based analysis can distinguish patients with and without DVT while providing image acquisition guidance for non-experts according to the clinical standard. Evaluation was performed on a sample size of *n* = 53 enroled patients from the same clinic, 34 DVT-positive patients and 30 additional patients from another clinic, *n* = 4 DVT positive. Algorithmic DVT diagnosis results in a sensitivity within a 95% CI range of (0.82, 0.94), specificity of (0.70, 0.82), the positive predictive value (PPV) of (0.65, 0.89), and a negative predictive value (NPV) of (0.99, 1.00).

Our method suggests a diagnosis based on robust segmentation in contrast to a direct image discriminator model. Consequently, our method does not rely on discrimination in the conventional ML sense. Our model learns predominantly from healthy volunteer data how a healthy vessel looks like and uses this knowledge to identify DVT suspected patients in the test data. This is different from traditional decision boundary modelling with fully supervised learning from a balanced dataset. Thus, our model is not noticeably affected by class imbalance issues in the training data; if the vein closes, the compression sequence is not DVT-suspected, otherwise it is. Identifying the correct vessel and interpreting the state of the vessel is the challenging part, which is addressed by our ML model. Data variability is relevant for the representation of the vessel itself. To improve this learnable variability, we use data augmentation as commonly used for image data^18^. All images are resampled to 150 × 150 pixels to facilitate real-time inference capabilities. We use image augmentation during training: random left/right flipping, ±15 pixel random translation, ±15^∘^ rotation, random zoom at a maximum factor of 0.05, intensity re-scaling with a maximum range of ±0.3.

ML has previously been studied for a variety of diagnostic approaches^19–21^. Several studies have applied ML in the context of VTE, although these ML applications have focused on developing CDSS that aid clinicians in VTE risk stratification of patients rather than diagnose VTE^12,22^. To the best of our knowledge, our work is a pioneering study that shows the potential benefits of ML for the diagnosis of DVT through imaging.

Our work evaluates all implications for the implementation of a ML model in a challenging clinical workflow like DVT diagnosis with ultrasound imaging, a pathway that requires direct human-machine interaction. This contrasts currently dominating ML methods for retrospective image analysis of tomographic data like CT or MRI, which usually presents itself to an algorithm clearly without imaging artefacts and in an often canonical orientation. Free-hand ultrasound poses additional challenges compared to these settings.

First, a user needs to be directed and guided to acquire images that are suitable to make a prediction through a ML model. This requires algorithmic provisions to discriminate useful images from images that do not adhere to a clinic standard. We solve this problem through training a discriminator ML model, which can identify predefined anatomical locations along the femoral vein.

Second, compression ultrasound requires the analysis of continuous image sequences which is challenging in a setup that requires real-time feedback. We solve this problem through a sliding window, multi-channel input approach, which enforces spatio-temporal consistency for a combined vein-segmentation with learned decision boundaries for identifying a vessel as fully closed. Furthermore, mobile ultrasound probes are used and connected to a GPU-accelerated laptop to provide sufficient computational power.

Third, image domain shift is a serious limitation of ML applications in healthcare. Domain shift occurs when a model is trained on images that have been acquired on one device while the testing is performed on images from other, previously unseen images from different devices. Commonly, a noticeable drop in performance is observed in such situations. We mitigate this problem through integrating image data from a diverse set of devices, covering almost the entire market for mobile ultrasound devices. Still, there is no established method for robust domain adaptation^23^. Hence, a risk of reduced performance remains when applying the presented algorithms to images from a new device. This risk must be avoided by deploying these algorithms exclusively with thoroughly tested, specific devices.

ML-supported devices such as described here are often summarised as clinical AI^21^. A critical element of any AI-based support tool is its clinical relevance.

DVT has a relatively low prevalence; 7.1% for a selected population who present to a DVT clinic as in our work and <0.003% in the general population. We factor this in when calculating PPV and NPV, thus providing values that are most informative for patients. In our case, a NPV of around 99% means that if the software-supported imaging test does not provide evidence for the presence of a blood clot, that there is an extremely low chance that this prediction is wrong and that the patient might still have DVT. Conversely, the PPV of our method is about 77% with a large 95% confidence interval of 12 percentage points. This means that if the automated imaging test gives a DVT positive result, that there is still a 20–30% chance that this diagnosis is wrong. This is addressed in possible clinical pathway integration strategies in Fig. 11. A positive test with AutoDVT will always lead to a confirmatory scan with an expert, who will also make treatment decisions which may include secondary criteria like for example the age of the thrombus. However, within the group who tested positive with AutoDVT, the expert’s chance of seeing an actual DVT-positive patient is more than 80%, which is notably higher than the current 7.1%. Increasing the pre-test probability for DVT will likely reciprocally increase the diagnostic utility and discriminatory power of the expert examination as well.

Literature and our own experiments show strong evidence that a DVT examination in primary care performed by non-experts is feasible. We would expect that rapid point of care diagnostics and wide availability of testing, which is conceivably enabled by our approach, would lead to timely treatment, decreased stress, and increased patient satisfaction. Furthermore, a cost analysis simulation model has been evaluated when integrating the proposed algorithm into the clinical practice. Assuming a willingness to pay £20 000/QALY^17^, a maximum NMB between £71 to £139 per examination could be attained when ML guidance is used by non-specialist workers for DVT diagnosis. This assumes zero costs for the use of the software; thus, it is the maximum achievable NMB. A DVT examination software tool could cost up to £72–£175 at the sensitivity and specificity levels measured in Table 5, before the NMB falls below £0. If the examination costs go above £72, then the conclusion that AutoDVT is cost-effective becomes more uncertain.

Our study has several limitations. First, in EVS1, we evaluate a prospectively enroled patient cohort retrospectively, on video sequences that have not necessarily been acquired at an optimal standard. Therefore, we had to curate the data and automatically extract clips from entire exam video recordings that would be most similar to clips as they would be acquired by the AutoDVT software guidance method. Furthermore, free-hand ultrasound examinations are highly operator-dependent, and every operator has a unique style of examination. Our proposed approach aims to standardise these styles to provide optimal input for subsequent image analysis parts and to aid clinical audits.

Second, standardised acquisition has been demonstrated in EVS2 but for both external validation sets, our patient cohorts are small, and we compare across-population with findings from literature. This limits the types of statistical techniques that can be employed in this study to evaluate statistical significance. We will soon start a multi-centre prospective trial that will address these issues to give further insights into the practical implications of employing AI support for DVT diagnosis. As suggested in the proposed diagnostic DVT decision trees (Fig. 11), the ultimate goal of employing AI support for DVT diagnosis would be to develop a ML-powered system using free-hand ultrasound that enables healthcare generalists at the point of care to exclude the presence of DVT in negative cases. If sufficient accuracy is achievable, this could obviate the need for a diagnostic scan performed by an expert user for DVT negative cases, leading to quicker diagnoses and further cost-benefits. Achieving this goal will require a number of clinical acceptance issues to be overcome. Perhaps the most important of these is the notion of clinical responsibility. When an expert user performs a scan, the presence or absence of DVT is determined by the expert user, who bears the clinical responsibility for the outcome of the test. By obviating the need for an expert user, the clinical responsibility and any associated liability must lie with the AI/ML-powered system and hypothetically associated teleradiology workers, since it is the system that determines the outcome of the test and not the non-specialist user holding the ultrasound probe. The implications of this are particularly significant in the context of a false negative outcome given the possibility of DVT progression to PE and even death. With this in mind, clinical acceptance is realistically only attainable if the AI/ML-powered system can achieve exceptionally high NPV, as shown in our work.

Hence, this study describes the first step of a larger clinical trial programme which we will use to ultimately evaluate the clinical efficacy of the AutoDVT software for the diagnosis of proximal DVT. The study we describe confirms that the AutoDVT software can diagnose DVT accurately. However, in order for the device to be accepted within the clinical community a large-scale efficacy study is required to confirm non-inferiority to expert-led compression US for proximal DVT diagnosis. Once this has been conducted, the device will be able to be offered as a diagnostic alternative to hospital clinic-based DVT diagnosis. In conclusion, our study shows the potential of a ML-powered system using free-hand ultrasound to identify DVT in clinical populations with high-throughput requirements and at the primary care level. Since access to ultrasound imaging is increasing and amplified through cost-effective mobile ultrasound devices, a ML-supported examination by less specialised front line care workers has the potential to be adopted for proximal DVT screening before confirmatory tests.

## Methods

### Study design

This study is a primary analysis of compression ultrasound scan recordings performed on prospectively enroled patients at the Oxford Haemophilia and Thrombosis Centre adult DVT clinic. The University of Oxford, UK, approved the study (Ethics: 18/SC/0220, IRAS 234007). All participants provided written informed consent. Eligible participants were consecutively recruited between January 2019 and December 2019. Patients were approached about participation in the study after their routine ultrasound DVT examination. After study information and consent, they were scanned for a second time by an expert radiographer. During the second scan a mobile ultrasound device was used (Philips Lumify L7). The examinations were recorded as mp4 videos. Patient identifying information has not been recorded in the videos but separately in a spreadsheet where it was tagged with a unique identifier (UID) by co-author Ch.D. Only the UID was used during downstream analysis.

A second pilot evaluation has been conducted in another clinic, the Ernst von Bergmann Klinikum Potsdam, Germany, (Ethics: S7(a)/2020). Eligible participants were recruited between November 2020 and April 2021. Patients were approached about participation and consented in the study after their routine ultrasound DVT examination. The examination was conducted with a Clarius L7 HD (2020) by a clinical expert. In contrast to the first data collection in Oxford, the AutoDVT software has been used by the operator in Potsdam for guidance and video acquisition.

In this work, we call the data set from the Oxford Haemophilia and Thrombosis Centre the EVS1 and data from Potsdam EVS2. Since the analysed prototype device is based on a ML computer algorithm, training data and preliminary testing data are required. Thus, preliminary data acquisition was performed on healthy volunteers (*n* = 246) and nine consenting patient volunteers who were examined for DVT (*n* = 4 DVT positive). The acquisition has been performed by two radiologists and three trained engineers. We call the data that is used for training of the model training set (Table 7). The volunteers and patients that have been left out from training to monitor the algorithm’s performance during development are collected in the internal validation set (Table 8).

**Table 7:** Training data overview.

Algorithm training data	Groin/thigh area model training	Knee area model training	Acquired data
Subjects	245	163	255
Number of compression sequences	1076	616	1500
*Annotated scan sequences*			
Background/no anatomical landmark or compression	169	169	169
LM0—external iliac vein	10	–	10
*Start of groin area after the inguinal ligament*			
LM1—Greater saphenous vein + common femoral vein at saphenofemoral junction	215	–	215
LM2—common femoral vein and artery	51	–	51
LM3—common femoral vein and superficial and deep femoral arteries	294	–	294
LM4—superficial and deep femoral veins and arteries	141	–	141
*End of groin area and beginning of thigh area at entrance to adductor canal*			
LM5—proximal thigh with superficial vein clearly visible with deep femoral vein clearly separated in deep tissue	123	–	123
LM6—mid thigh with superficial femoral vein and artery in the adductor canal	288	–	288
LM7—distal thigh, same anatomy as LM6	2	–	2
*End of thigh area and beginning of knee area*			
LM8—proximal popliteal area, with popliteal vein and artery	–	130	130
LM9—middle popliteal area, with tibial-fibular trunk and popliteal artery	–	141	141
LM10—distal popliteal area, with anterior and posterior tibial and fibular veins and popliteal artery	–	186	186
Total number of manually annotated images	111,546	88,823	167,145

Subjects may contain more than one landmark; thus, subject IDs may be present in the training and internal validation set. Individual sequences are either in one or the other set. Landmarks used for the groin model in this study are LM0–LM4 and those for the knee area are LM8–LM10.

**Table 8:** The internal validation set represents a random 10% split at the subject level of the overall available training data.

Internal validation data	Groin/thigh area validation sequences	Knee area validation sequences	Acquired internal validation sequences
Total subjects	25	17	26
Number of compression sequences	88	58	133
*Annotated scan sequences*			
Background/no anatomical landmark or compression	13	13	13
LM0—external iliac vein	–	–	–
*Start of groin area after the inguinal ligament*			
LM1—Greater saphenous vein + common femoral vein at saphenofemoral junction	17	–	17
LM2—common femoral vein and artery	1	–	1
LM3—common femoral vein and superficial and deep femoral arteries	27	–	27
LM4—superficial and deep femoral veins and arteries	11	–	11
*End of groin area and beginning of thigh area at entrance to adductor canal*			
LM5—proximal thigh with superficial vein clearly visible with deep femoral vein clearly separated in deep tissue	10	–	10
LM6—mid thigh with superficial femoral vein and artery in the adductor canal	25	–	25
LM7—distal thigh, same anatomy as LM6	–	–	–
*End of thigh area and beginning of knee area*			
LM8—proximal popliteal area, with popliteal vein and artery	–	20	20
LM9—middle popliteal area, with tibial-fibular trunk and popliteal artery	–	11	11
LM10—distal popliteal area, with anterior and posterior tibial and fibular veins and popliteal artery	–	16	16
Total number of manually annotated images	9598	8257	15,523

Landmarks used for the groin model are LM0–LM4 and those for the knee area are LM8–LM10.

**Fig. 7:**
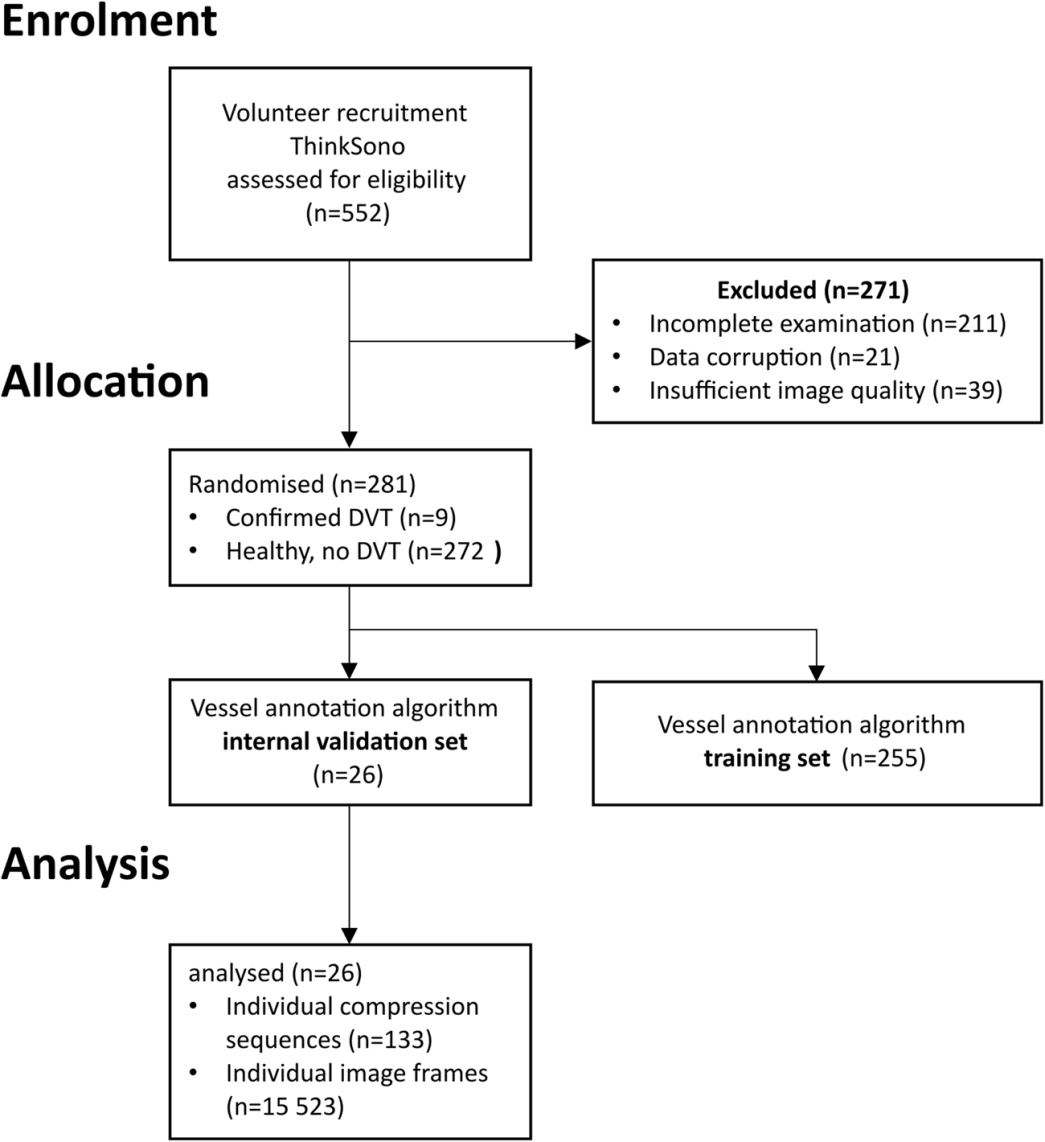
Consort diagram for inclusion of volunteer scans into the training set and internal validation set. Dataset curation for the training and internal validation data. Our approach can be trained from image data that originates predominantly from healthy volunteers.

The ML model’s task is to annotate vessels, find anatomical landmarks, and analyse vessel compression state automatically. DVT diagnosis is done by automatization of the standard clinical ultrasound compression algorithm in a heuristic computer programme, based on the biometrics acquired from the ML model during the scan. Thus, the ML model has been trained mainly on data from healthy volunteers (*n* = 246, age range 18–84, BMI < 30) and compression sequences from consented patients with confirmed DVT (*n* = 9). An overview over the inclusion criteria is given in Fig. 7 and the training data population in Table 7. An overview over the internal validation set is shown in Table 8. All compression sequences have been manually annotated (marking pixels that belong to vein or artery by different colour labels) by a trained workforce (*n* = 23 trained labellers) including medical students and employees of ThinkSono Ltd to (a) train the algorithm and (b) evaluate its performance quantitatively.

Image quality control has been performed by a medical student according to a specialist-defined scheme.

#### Quality control scoring system

We use a 10-point expert image quality scoring as it is outlined below to curate video data that has not been acquired under AutoDVT guidance and real-time quality control. The quality cut-off, i.e., the minimum required quality has been less or equal to a total score of 20 in this study.

1. Vessel boundaries in video frame images
  - Fully in Frame: 1
  - Cut off <50% of vessel size during parts sequence: 2
  - Cut off <50% of vessel size during entire sequence: 3
  - Cut off >50% of vessel size during parts of sequence: 4
  - Cut off >75% of vessel size during part of sequence or >50% over entire sequence: 5
2. Adherence to regular LM configuration:
  - Strongly adherent to LM configuration: 1
  - Different positions of veins and arteries than regular configuration, mix of LMs: 2
  - Loss of LM position for parts of sequence: 3
  - Additional/missing (large) vessels for LM: 4
  - Additional/missing vessels and different positions: 5
3. Contrast of veins to tissue
  - Dark veins, bright tissue: 1
  - Somewhat contrasted veins: 2
  - Veins and tissue almost same echogenity: 3
4. Contrast of arteries to tissue:
  - Dark arteries, bright tissue: 1
  - Somewhat contrasted arteries: 2
  - Arteries and tissue almost same echogenity: 3
5. Sharpness of vein boundaries
  - Clear boundaries with strong dorsal echo amplification: 1
  - Well discernible boundaries with some dorsal echo amplification: 2
  - Poorly visible boundaries without echo amplification: 3
6. Sharpness of artery boundaries
  - Clear boundaries with strong dorsal echo amplification: 1
  - Well discernible boundaries with some dorsal echo amplification: 2
  - Poorly visible boundaries without echo amplification: 3
7. Overall gain of image
  - Medium gain range, good quality: 1
  - Image too bright: 2
  - Image too dark: 3
8. Depth of image
  - Image ends about 1 cm below lowest vessel: 1
  - Image ends within 1–2 cm below lowest vessel: 2
  - Image ends 2+ cm below lowest vessel: 3
9. Image artefacts
  - Good quality, only minor artefacts: 1
  - Multiple smaller artefacts, also in/over the vessels: 2
  - Large image problems, i.e., probe not fully on leg: 3
10. Probe Movement in sequence
  - Medium paced compression and decompression, no lateral or horizontal movements, full vein compression: 1
  - Very fast or very slow compression and decompression, no lateral or horizontal movements, incomplete compression on healthy veins: 2
  - Minimal lateral/horizontal movement: 3
  - Lots of movement: 4

Total: 10–35 Points

### Ultrasound protocol

**Fig. 8:**
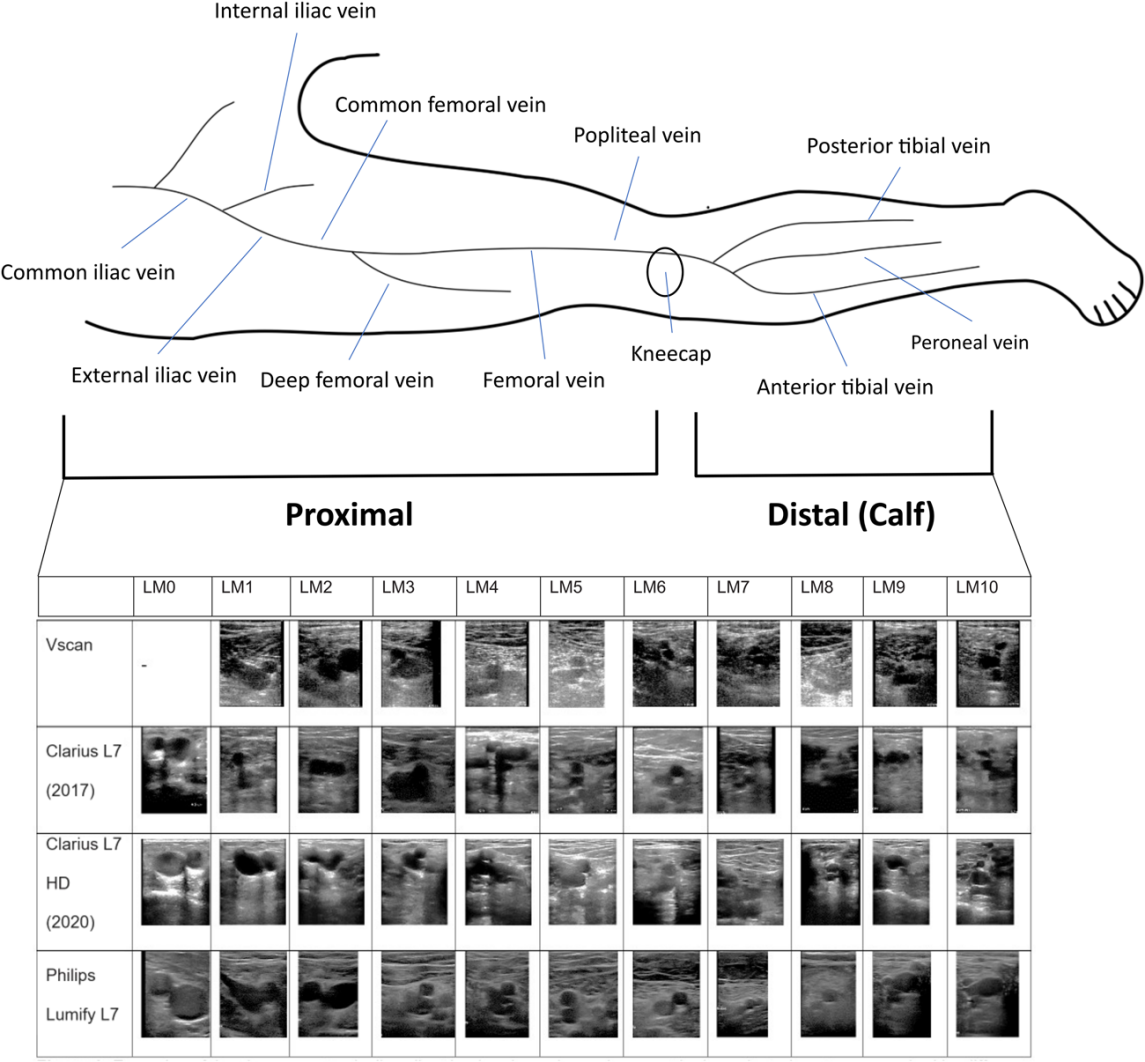
Examples of the chosen anatomically salient landmarks and overview over the investigated anatomy. Images have been acquired by different acquisition devices and from different subjects. This figure illustrates the diversity in our dataset. See the overview above the table and Tables 7 and 8 for a description for the location of these landmarks. These example images have been manually cropped and contrast normalised for better readability.

Non-enhanced ultrasound imaging was performed by a research physician or radiologist (at least one year of hands-on ultrasound DVT imaging training) using either Clarius L7 (2017) and Clarius L7 HD (2020) or Philips Lumify L7 or GE VScan Extend (scanned with linear probe, only for training data) ultrasound devices. Example images for these scanners are shown in Fig. 8. Two-point compression ultrasound was used for this study. Clinically, a compression is deemed adequate when the vein is compressed fully. A vein that does not compress at the same pressure, at which a healthy vein would collapse, indicates DVT. The femoral vessels were examined from 2 cm distal to the saphenofemoral junction to 2 cm proximal from the inguinal band. The superficial femoral vessels were examined in the adductor canal. The examination of the popliteal vein starts from the distal 2 cm of the popliteal vein and its trifurcation into the anterior tibial vein, posterior tibial vein, and the peroneal vein. The entire examination has been recorded as screen capture videos, cropped to the ultrasound image area without user-interface content and resampled with bilinear interpolation to 150 × 150 pixels. Participants were positioned in a supine position, with the hip rotated outwards by about 60–80^∘^ and the knee flexed at about 60^∘^. The knee area was examined either supine with neutral hip and knee flexed at 80–90^∘^ or sitting upright with knee hanging loose over the gurney edge at 90^∘^.

### Statistical analysis

Ultrasound has a sensitivity of 94% and a specificity of 97% for DVT detection^24,25^ when performed by specialised radiologists. Two studies reported sensitivity of 84.4–90.0% and specificity of 97.0–97.1% when intensely trained nurses and GPs were the ultrasound operator^9,10^. This means that there is strong evidence that non-specialists like nurses and GPs can (a) acquire ultrasound imaging data of high enough quality for diagnosis and (b) that these operators are able to correctly identify DVT.

#### Sample size

The sample size in this manuscript is 167,145 annotated ultrasound images for model training from 255 volunteers and 15,523 annotated images from 26 participants for internal model validation. For prospective model evaluation we use 19,134 images in 121 video recordings from 53 patients, 34 DVT positive, in EVS1, and 18,399 images in 150 compression video clips from 30 patients, four DVT positive, in EVS2. This provides 83 patient observations for algorithm evaluation that have not been part of the algorithm training data. On the patient level, sample sizes like this are in line with other studies evaluating algorithmic diagnostic decision support. Some recently reported external validation sample sizes of, e.g., 50^26,27^, 91^28^, and 198^29^ subjects during retrospective testing and 80^30^ to 97^31^ during prospective testing. Statistical approximations for sample size estimation^32^ suggest that eight patients with the required outcome would be sufficient in an external validation set, given an incident rate of 7.1% (Table 1) as observed in our UK thrombosis clinic. Combining EVS1 and EVS2, we have included 38 patients with confirmed DVT and 45 patients who were suspected but did not suffer from DVT. This provides an almost balanced dataset for testing. For the training of our model, a balanced dataset with respect to diagnostic outcome is not required. The core algorithm focuses on accurate vessel segmentation, which can be learned from compression ultrasound imaging from healthy volunteers with manual delineations of the visible vessels. Diagnosis is suggested indirectly through the compressibility of the vein. We provide fourfold cross-validation results on the individual sets (80%:10%:10% training:validation:testing with non-overlapping data splits). This allows to provide ~95% confidence intervals for the core algorithm’s performance for the vessel segmentation and anatomical guidance tasks. The power of this study is above 0.8 at a significance level of 0.05, with a Cohen’s d effect size of 0.5, when assuming an effect between 0.9 (without software support *n*^9 ^= 697, *n*^10 ^= 1107) and 0.95 (with software support, this study *n* = 53, *n* = 30) with a standard deviation of 0.1. For this setting, 51 patients are required as a minimum to reach a power of 0.8. We achieve this for EVS1 alone (*n* = 53) and for the combined analysis of EVS1 with EVS2 (*n* = 83).

The R software package (©The R Project for Statistical Computing) has been used for numerical power analysis.

Algorithms are evaluated at the participant level. To evaluate classifier performance, we calculate sensitivity, specificity, PPV, NPV, and overall diagnostic accuracy for DVT identification for the internal and external validation sets.

We also generate the ROC of the DVT classification score for the external validation sets and calculated the area under the ROC (AUC). We show confusion matrices at the optimal algorithm threshold.

### Algorithm design

This study aims to validate the effectiveness of an ML-powered device (AutoDVT) for the diagnosis of proximal DVT. AutoDVT is a CE-marked software product (93/42/EEC 40873) that is coupled to a handheld CE-marked ultrasound machine. The AutoDVT software has two functions: (1) Directing the user to correctly position the ultrasound to complete a thorough scan, and (2) analysing the scan results to confirm the presence/absence of a thrombus.

The software uses a fully automated ML vessel segmentation network with auxiliary branches that predict the anatomical location of the ultrasound image relative to the deep veins in the leg and the compression status of the vein (open or closed). Veins have been labelled by a radiologist to be either open or closed and fully compressed. Two networks with identical design/architecture have been trained: one for the groin/thigh area and one for the knee area. The subject IDs overlap between the training set and internal validation set because a sequence can have multiple landmarks but belong to either a healthy patient or a patient with confirmed DVT. See Table 7 for an overview over the algorithm training data and Table 8 for the internal validation data. Annotations include manual delineations of vein and artery cross sections in the images as well as discrete image-level labels for eleven anatomical locations. To facilitate algorithmic evaluation, we have defined anatomically salient landmarks (LM0–LM10) on the common femoral vein, superficial femoral vein, and popliteal vein. Example images for these landmarks, acquired with the different ultrasound probes that are used for algorithm training in this study, are shown in Fig. 8.

To exclude DVT an operator must follow a protocol as instructed by the software. This protocol resembles the clinical practice of three-point or two-point examinations^33–35^, which means doing compression ultrasound in two to three regions where the greatest risk of developing thrombosis occurs. For three-point compression protocols, these regions include: (1) the common femoral vein at the level of the inguinal crease (LM0–LM4), (2) the superficial femoral vein superior in the adductor canal (LM5–LM7), and (3) the popliteal vein and its trifurcation in the popliteal fossa (LM8–LM10).

For two-point compression protocols the same regions are examined except (2), i.e., LM0–LM5 in the groin and LM8– LM10 in the knee. To maximise the overlap between common procedures in the clinics from where our external validation sets originate, we investigate in this study the effectiveness of algorithmically evaluated two-point compression DVT examinations.

Thus, using the training set, the discriminator parts in the ML models are trained on consolidated groups of landmarks LM0–LM1, LM2–LM3–LM4, i.e., two groups, for (1) and one group, LM8–LM9–LM10, for (3). This means three successful vein compressions, two in the groin area and one in the knee area, are required in total to exclude DVT. All identified anatomical locations must show fully compressible veins, otherwise the participant is categorised as suspected DVT case.

**Fig. 9:**
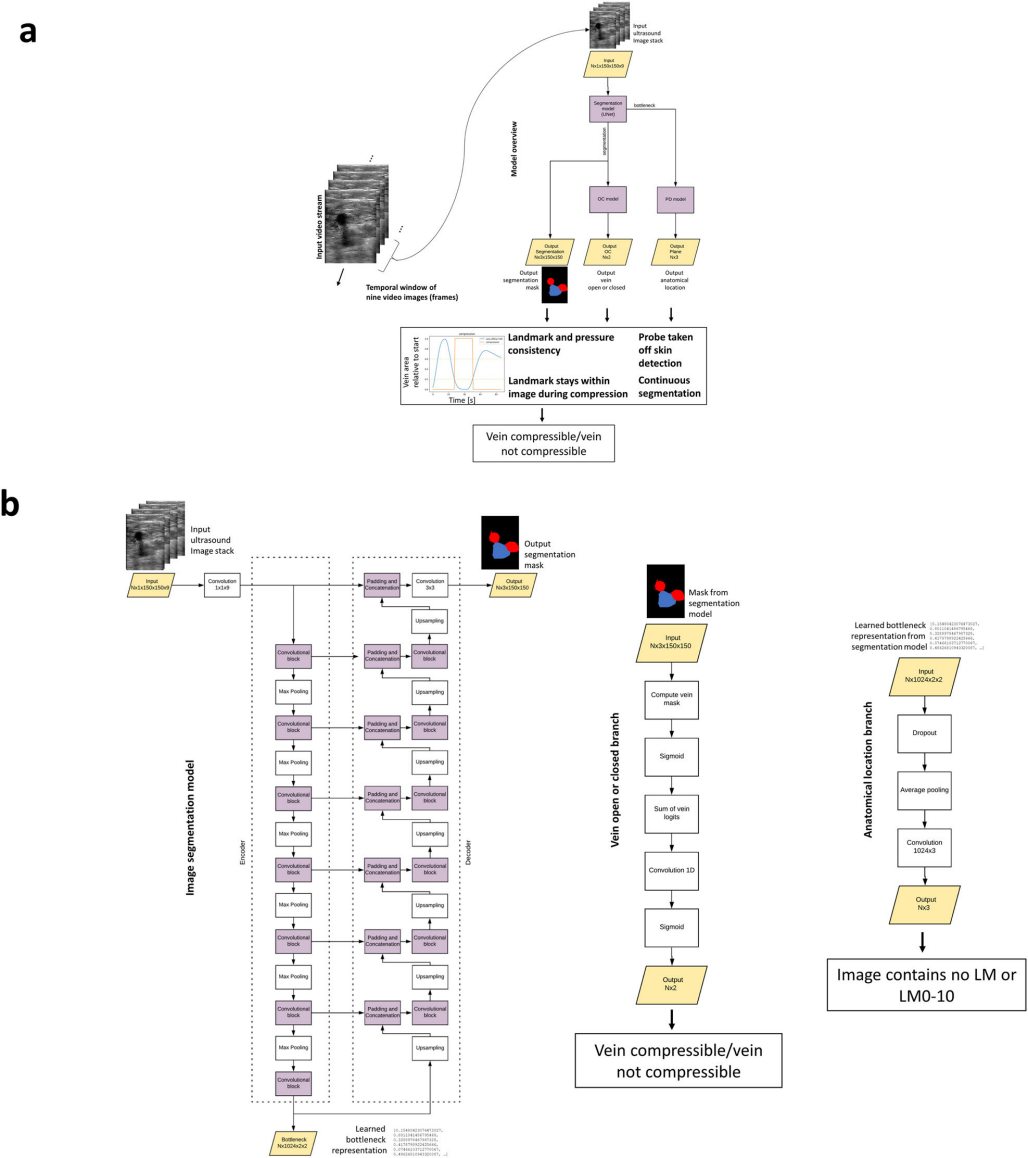
Overview over the AutoDVT prototype core algorithm. **a** whole overview and **b** overview over the individual branches. A U-Net^41^ serves as a backbone for automatic delineation of vein and arteries (**b**). The prediction of the anatomical location of the image is based on our previous work^15^. Network branches predict the anatomical location and whether the vessel is open or closed under pressure. Landmark predictions are performed from the learned numeric representation in the bottleneck layer; vessel compression state is predicted from the output segmentation mask. The network components are connected and can be trained through back-propagation^42^ in an end-to-end manner. The input is a stack of nine images (individual video frame images resampled to 150 × 150 pixels) from an ultrasound video stream that moves by one in a sliding window fashion. A single segmentation mask is produced for the last-most image within approximately 25 ms. Two separate models with identical architecture are trained, one for the groin area (LM0–LM5) and one for the knee area (LM8–LM10). Each model holds 31,475,527 parameters. (OC = open/close).

Two deep ML networks with identical architecture as shown in Fig. 9 were trained on a GPU server (Nvidia Tesla K80) using the Adam optimizer with momentum 0.9 to optimise the parameters of the network. Binary cross entropy (BCE, Eqs. (1) and (2)) is used for the segmentation task (one-hot encoded) and the vein open/closed task. Cross entropy (CE, Eq. (3)) is used for the anatomical landmark detection task as an error metric.

1 $${{{{\mathcal{L}}}}}_{\mathrm{segmentation}(\mathrm{one-hot-encoded})}=-\frac{1}{N}\mathop{\sum }\limits_{i=1}^{\mathrm{pixels}}{y}_{i}{{\mathrm{log}}}\,(p({y}_{i}))+(1-{y}_{i}){{\mathrm{log}}}\,(1-p({y}_{i}))$$

2 $${{{{\mathcal{L}}}}}_{\mathrm{anatomicallocation}(\mathrm{one-hot-encoded})}=-\frac{1}{N}\mathop{\sum }\limits_{i=1}^{11}{y}_{i}{{\mathrm{log}}}\,(p({y}_{i}))+(1-{y}_{i}){{\mathrm{log}}}\,(1-p({y}_{i}))$$

Where *y* is the real label and *p*(*y*) is the predicted probability for the image belonging to this label.

3 $${{{{\mathcal{L}}}}}_{(\mathrm{veinopenorclosed})}=-(y{{\mathrm{log}}}\,(p(y)))+(1-y){{\mathrm{log}}}\,(1-p(y))$$

The total error metric (loss function) for our network results as

4 $${{{{\mathcal{L}}}}}_{\mathrm{total}}=\alpha {{{{\mathcal{L}}}}}_{\mathrm{segmenation}}+\beta {{{{\mathcal{L}}}}}_{\mathrm{anatomicallocation}}+\gamma {{{{\mathcal{L}}}}}_{\mathrm{veinopenrclosed}},$$

where *α* and *β* are adjustable hyper parameters. We use *α* = 100 and *β* = *γ* = 1. The PyTorch deep learning framework^36^ has been used for our implementation. A series of manually tuned temporal quality control functions ensure robust communication with the user regarding vessel location in the image, quality of compressions, imaging parameters and placement of the probe (Fig. 9).

The internal validation set (*n* = 26 healthy subjects, held out from training) has been used to test the models’ performance during development by comparing segmentations to manual delineations of the vessels and manual, categorical image labels with respect to the anatomical locations (LM0–LM10) and the vessel compression status (open or fully closed). For categorical labels, the F1-score is used,

5 $$F1=\frac{\,{{\mbox{true positive classifications}}}}{{{\mbox{true positive classifications}}}+\frac{1}{2}({{\mbox{false positive classifications}}}+{{\mbox{false negative classifications}}}\,)}$$

And for segmentation masks the Sørensen-Dice Coefficient is applied per label (background, artery, vein),

6 $${DICE}=\frac{2\times \,{{\mbox{true positive pixels}}}}{2\times {{\mbox{true positive pixels}}}+{{\mbox{false positive pixels}}}+{{\mbox{false negative pixels}}}\,}$$

In addition, the bounding boxes for the individual segmentation masks are generated and the intersection over union (IoU = Jaccard index) is computed, which is a common performance metric for object detection tasks,

7 $${IoU}=\frac{F1}{(2-F1)}=\frac{\,{{\mbox{Area of overlap with true bounding box}}}}{{{\mbox{Area of union with true bounding box}}}\,}$$

**Fig. 10:**
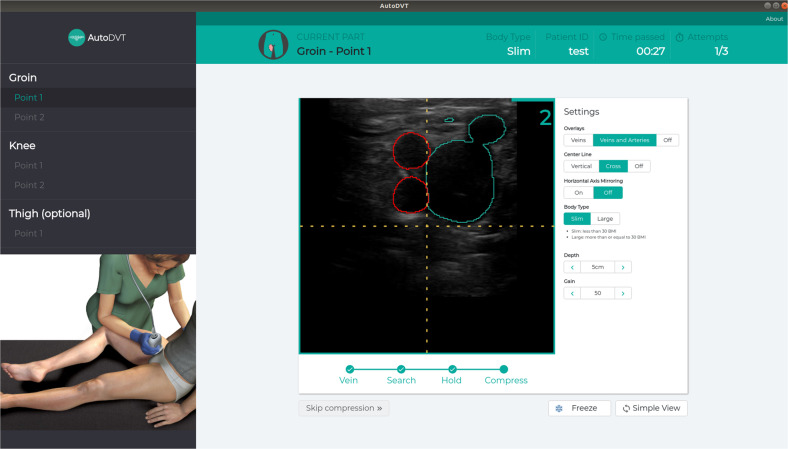
Prototype implementation user interface. The AutoDVT software instructs users to locate a given landmark, instructs to perform a correct compression and evaluates the result automatically.

In an end-user scenario a non-expert operator would have three to five attempts to complete a compression, otherwise referral is recommended. A screenshot of the AutoDVT software during use is shown in Fig. 10. During our experiments, all compressions have been competed in under five attempts.

#### Technical uniqueness of the proposed framework

We propose a triple-task convolutional neural network (CNN) fully integrated into a clinical prototype device that jointly classifies the anatomical landmark plane in the current field-of-view, scores vein compressibility and provides semantic segmentation masks for arteries and veins. The proposed network architecture can intrinsically learn to interpret video data to perform localisation, segmentation, local deformation estimation and classification from weak discrete labels that characterise whole images, e.g., anatomical landmark locations. Furthermore, it is designed to require a reasonably low number of floating-point operations to facilitate real-time performance.

**Fig. 11:**
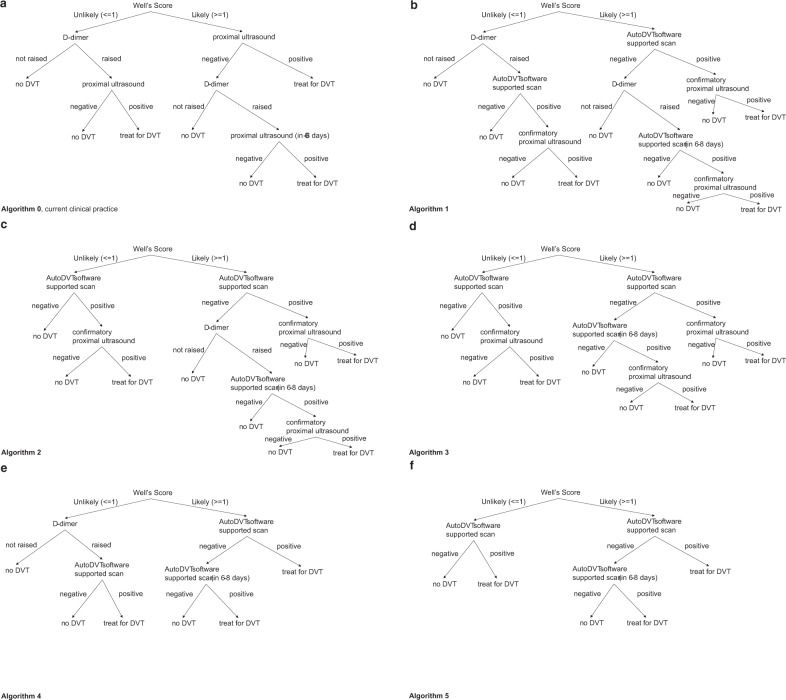
Possible integration strategies for our approach into DVT diagnostics pathways. **a** current clinical algorithm to diagnose DVT without software support according to UK NICE guidelines^6^ and **b**–**f** possible variants to integrate ML software support into the clinical pathway. Algorithms 1–3 shown in (**b**–**d**) generate a positive net monetary benefit (cf. Fig. 6). The examined modifications have been suggested by health economics and clinical experts. Note that treatment options may further depend on the age of the clot, which might be manually estimated during confirmatory ultrasound scans^43^.

### Cost effectiveness

We simulated the potential cost-effectiveness of a ML-enabled approach at the front line of care, where non-specialists may perform the examination independently. A decision tree analytic model was designed and implemented in Microsoft Excel (©Microsoft Corporation) to estimate the lifetime costs and benefit measured in terms of quality-adjusted life years (QALYs) for different proximal DVT testing algorithms. The current clinically used diagnostic DVT algorithm is shown in Fig. 11a and possible integration strategies for our method are shown in Fig. 11b–f.

The cost analysis model adheres to guidelines issued by the National Institute of Health and Care Excellence (NICE)^6^. It uses an UK NHS and personal social services perspective with costs at 2018/19 prices and with discounting for both costs and QALYs being undertaken at 3.5% per annum. Note that costs associated with tangible and intangible expenses that families can incur in the event of disability or even death due to misdiagnosis would not be considered by either NHS or personal social services expenditure and is commonly excluded from a NICE appraisal ^6^.

The model uses sensitivity (the ability of a test to correctly identify a patient with a true proximal DVT) and of specificity (the ability of a test to correctly identify a patient without a true proximal DVT) as measured on the external validation sets in this study. We also include clinical tests (Wells Score, D-dimer, and proximal ultrasound) that form part of the diagnostic algorithm.

Our cost analysis model splits patients into two subgroups at the start of each algorithm, a subgroup in which patients have a proximal DVT and a subgroup in which patients do not have a proximal DVT. Measured sensitivity and specificity values are used alongside an estimate of the prevalence of proximal DVT of 14.7% taken from Kilroy et al.^37^ to estimate the number of patients (from a cohort of user specified size) that receive each clinical test and their ultimate diagnoses (proximal DVT or not). Patients with a diagnosed proximal DVT will receive treatment. We generate four possible outcomes for patients based on their DVT status and the results of each diagnostic algorithm: Treated patients with a true DVT (true positive patients), treated patients without a true DVT (false positive patients), untreated patients without a true DVT (true negative patients) and untreated patients with a true DVT (false negative patients). Each of the four diagnostic accuracy outcomes have estimated associated costs incurred and utility accrued for the patients. These numbers are multiplied by the proportion of patients in each outcome and are combined with the costs of each test to obtain estimates of the total costs and QALYs for the diagnostic algorithm. When costs and QALYs are obtained for diagnostic algorithms with and without the ML model, the estimated incremental cost-effectiveness ratio for AutoDVT can be calculated.

#### Parameters for the cost-effectiveness model

Test characteristics have been taken from Goodacre et al.^24^ and are presented in Table 9 with the statistical distributions used in stochastic analysis presented in Tables 10–13.

**Table 9:** Efficiency parameters for each test.

Description		Mean
Wells Score: Proportion of patients with a proximal DVT characterised as:		
	High risk	0.68
	Moderate risk	0.25
	Low risk	0.07
Wells Score: Proportion of patients without a proximal DVT characterised as:		
	High risk	0.11
	Moderate risk	0.41
	Low risk	0.48
D-dimer (assumed to be an enzyme-linked immunosorbent assay (ELISA) test)		
	Sensitivity for proximal DVT	0.98
	Specificity for proximal DVT when	
	Wells Score is high risk	0.34
	Wells Score is moderate risk	0.45
	Wells Score is low risk	0.52
Proximal ultrasound		
	Test sensitivity	0.95
	Test specificity	0.94

**Table 10:** Test Efficiency Well’s test.

Description	Mean	Distribution	Parameter 1	Source
Proportion of patients with proximal DVT characterised as high risk	0.68	Dirichlet	105.61	^17^
Proportion of patients with proximal DVT characterised as moderate risk	0.25	Dirichlet	38.83	^17^
Proportion of patients with proximal DVT characterised as low risk	0.07	Dirichlet	10.87	^17^
Proportion of patients without proximal DVT characterised as high risk	0.11	Dirichlet	40.78	^17^
Proportion of patients without proximal DVT characterised as moderate risk	0.41	Dirichlet	151.99	^17^
Proportion of patients without proximal DVT characterised as low risk	0.48	Dirichlet	177.94	^17^

**Table 11:** Test Efficiency D-dimer test.

Description	Mean	Distribution	Param. 1	Param. 2	Source
Sensitivity for proximal DVT	0.98	Beta	736.91	15.04	^17^
Specificity for proximal DVT— Wells’s outcome: high risk	0.34	Fixed			^17^
Specificity for proximal DVT— Wells’s outcome: moderate risk	0.45	Beta	4278.13	5228.83	^17^
Specificity for proximal DVT— Wells’s outcome: low risk	0.52	Fixed			^17^

**Table 12:** Proximal DVT prevalence and outcomes associated with treated and untreated proximal DVT.

Description		Mean	Distrib.	Param. 1	Param. 2	Source
Proximal DVT prevalence		0.15	Beta	41	238	(37 and Table 1)
Treated proximal DVT						
	Probability of fatal pulmonary embolus	0	Beta	17	4204	^17^
	Probability of non-fatal pulmonary embolus	0.01	Beta	33.4	4070.6	^17^
	Probability of post thrombotic syndrome	0.05	Beta	28	500	^17^
Outcomes associated with warfarin treatment						
	Fatal haemorrhage	0	Dirichlet	a = 37		^17^
	Non-fatal intracranial haemorrhage	0	Dirichlet	b = 13		^17^
	Non-fatal non-intracranial haemorrhage	0.02	Dirichlet	c = 226		^17^
	No haemorrhage	0.98	Dirichlet	d = 10.481		^17^
Untreated proximal DVT						
	Probability of fatal pulmonary embolus	0.02	Beta	5	263	^17^
	Probability of non-fatal pulmonary embolus	0.09	Beta	25	243	^17^
	Probability of post thrombotic syndrome	0.33	Beta	5.21	10.57	^17^

**Table 13:** QALYs associated with outcomes and QALYs accrued by patient category.

Description		Mean	Distribution	Param. 1	Param. 2	Source
Normal age-specific discounted quality-adjusted life expectancy (QALYs)		11.6	Fixed			^17^
Lifetime utility multiplier associated with						
	Non-fatal pulmonary embolus	0.94	Beta	19.43	1.24	^17^
	Non-fatal intracranial haemorrhage	0.29	Beta	8.34	20.41	^17^
	Post thrombotic syndrome	0.98	Beta	232.64	5.48	^17^
Lifetime QALY’s accrued by						
	Patients with a DVT who are treated	11.5	[A]			
	Patients with a DVT who are untreated	11.2	[A]			
	Patients without a DVT who are treated	11.5	[A]			

[A]: The variance on these parameters is based on the variance of the parameters that make up these values.

Treatment reduces the probability of a patient with a DVT experiencing a fatal or non-fatal pulmonary embolism (PE) or post-thrombotic syndrome (PTS). However, treatment is associated with risks of fatal haemorrhage, non-fatal intracranial haemorrhage, and non-fatal non-intracranial haemorrhage.

According to Goodacre et al.^24^ patients who do not experience any of a PE, PTS or a haemorrhage accrue a mean of 11.58 discounted lifetime QALYs. Mean quality of life multipliers for PTS, non-fatal PE and non-fatal intracranial haemorrhage of 0.977, 0.94, and 0.29 respectively were also presented by Goodacre et al.^24^ with statistical distributions used in the stochastic analysis presented in Tables 10–13. These data were used to estimate total QALYs for the four diagnostic accuracy outcomes.

The lifetime, discounted, QALYs accrued by patients in each classification differ based on their true DVT status and their results from each diagnostic algorithm. Untreated patients with a DVT remain at high risk of PE and PTS but do not have the risks of haemorrhage associated with treatment. Treated patients with a DVT have reduced risks of PE and PTS but have the risk of haemorrhage associated with treatment. Treated patients without a DVT have the same risk of PE and PTS as the general population but are subject to the risks of haemorrhage associated with treatment. Untreated patients without a true DVT will accrue the same discounted lifetime QALYs as the general population. The QALYs accrued in each of the four diagnostic accuracy outcomes are shown in Table 14.

**Table 14:** Estimated QALYs accrued, and costs incurred for each diagnostic accuracy outcome.

Description	QALYs accrued	Cost incurred [UK £]
True Positive (DVT – treated)	11,464	1321
False Negative (DVT – untreated)	11,207	2214
False Positive (No DVT – treated)	11,530	975
True Negative (No DVT – untreated)	11,580	0

The discounted lifetime costs associated with patient outcomes were taken from^24,38^. Where appropriate costs were uplifted to 2018/19 values using inflation indices presented in Curtis et al.^38^.

The lifetime costs associated with PTS and non-fatal intracranial haemorrhage were both composite costs including the cost of a first attendance at a vascular surgery outpatient clinic and the cost of subsequent vascular surgery outpatient clinics visits for PTS and the cost of care in the first year and subsequent years in the case of non-fatal intracranial haemorrhage. The total cost associated with PTS and the method used to calculate these were included in Goodacre et al.^24^ together with the costs of the components of the total cost. From this, it was estimated that the expected lifetime of patients with PTS was 11.67 years. No such information was provided for patients experiencing a non-fatal, non-intracranial haemorrhage and thus it was assumed that the same expected lifetime applied when calculating costs.

Treatment for DVT consists of approximately eight days of low molecular weight (LMW) heparin followed by ninety days of Warfarin. The total cost of DVT treatment of £845 is estimated using the same derivation as that used in^24^ with one change: The current version of the British National Formulary^39^ indicates that the initial dose of LMW heparin in the treatment of DVT is a large loading dose with subsequent smaller maintenance doses, thus the initial loading dose will be associated with a greater cost than subsequent maintenance doses. The costs of LWM heparin and warfarin are taken from the current version of the British National Formulary^39^. Additional resource use such as GP visits and anticoagulant clinic visits and their unit costs^24,38^ and NHS Reference Costs 2015–2016^40^, where appropriate costs have been inflated to 2018/19 values using inflation indices presented by Curtis et al.^38^. The costs associated with outcomes associated with DVT or with treatment for DVT are shown in Table 15.

**Table 15:** Costs associated with outcomes of DVT and complications associated with the treatment of DVT.

Description	Mean [UK £]
Treatment of fatal PE	1650
Treatment of non-fatal PE	1601
Lifetime treatment of PTS	4663
Fatal intracranial haemorrhage	9334
Lifetime treatment for non-fatal intracranial haemorrhage	64,147
Non-fatal non-intracranial haemorrhage	805

The costs associated with each diagnostic test have been taken from Goodacre^24^ and the NHS^40^ and are presented in Table 16.

**Table 16:** Costs of the diagnostic tests.

Description	Mean [UK £]	Distribution	Parameter 1	Parameter 2	Source
Well’s test	9.66	Gamma	25	0.34	^17^
D-dimer	19.5	Gamma	25	0.78	^16^
Proximal ultrasound	77.19	Gamma	319.23	0.25	^40^

## Data Availability

Algorithm raw performance results of this study are available from the corresponding author upon reasonable request. Image data access in line with the informed consent of the participants, subject to approval by the project ethics boards and under a formal Data Sharing and License Agreement on a case-by-case basis. The data are not publicly available due to them containing information that could compromise research participant privacy/consent. Qualified researchers can apply for access via email to https://thinksono.com/ (info@thinksono.com).

## Code availability

Custom computer software (AutoDVT) paired with ultrasound acquisition devices can be licensed from ThinkSono Ltd (hello@thinksono.com). Data analysis code in R, Microsoft Excel*Ⓡ*, and Python and model structure in PyTorch format can be requested from the corresponding author.
